# Impact of Alcohol Intake on Skeletal Muscle: A Large Cross-Sectional Analysis in Japanese Adults

**DOI:** 10.3390/nu17050894

**Published:** 2025-03-03

**Authors:** Masahiro Matsui, Akira Fukuda, Saori Onishi, Kosuke Ushiro, Tomohiro Nishikawa, Akira Asai, Soo Ki Kim, Hiroki Nishikawa

**Affiliations:** 1 Second Department of Internal Medicine, Osaka Medical and Pharmaceutical University, Takatsuki 569-8686, Osaka, Japan; 2 Osaka Medical and Pharmaceutical University Health Science Clinic, Takatsuki 569-8686, Osaka, Japan; 3 Department of Gastroenterology, Kobe Asahi Hospital, Kobe 653-8501, Hyogo, Japan

**Keywords:** alcohol, heavy drinking, skeletal muscle, fat mass, large clinical study

## Abstract

Aims: To clarify the impact of alcohol intake on skeletal muscle mass (SMM) using data from Japanese health checkup recipients (8405 males and 11,509 females). The fat-free (FF) index was regarded as the FF mass divided by height squared (kg/m^2^). Methods: The subjects were classified into four groups (type A (never drinker), B (chance or mild drinker), C (moderate drinker), and D (severe drinker)) according to the amount of alcohol consumed. Results: The average age in males and females was 52.2 and 50.1 years, respectively (*p* < 0.0001). The average FF index in males and females was 18.5 and 15.1 kg/m^2^, respectively (*p* < 0.0001). The proportion of subjects of type A, B, C, and D was 36.5%, 44.2%, 5.9%, and 13.4%, respectively, in males, and 59.8%, 31.3%, 3.7%, and 5.1%, respectively, in females (*p* < 0.0001). The average FF index in type A, B, C, and D males was 18.43, 18.62, 18.12, and 18.16 kg/m^2^, respectively (overall *p* < 0.0001). The average FF index in type A, B, C, and D females was 15.17, 15.14, 15.15, and 14.78 kg/m^2^, respectively (overall *p* < 0.0001). Conclusions: Habitual heavy drinking has a negative effect on SMM. However, from the standpoint of maintaining SMM, it is not necessary to completely abstain from alcohol.

## 1. Introduction

In Japan, alcoholic beverages have been consumed at festivals, dinners, and many other occasions since ancient times and have become an established and familiar part of daily life and culture [1]. However, alcohol consumption is associated with various problems in terms of health maintenance. In Japan, alcohol consumption is declining as the total population declines and age, and the percentage of male drinkers has declined slightly, while the percentage of heavy drinkers is increasing [2]. It is estimated that in Japan, there are more than 10 million habitual drinkers with increased risk of lifestyle-related diseases and more than 1 million alcohol-dependent patients, but the reality is that only about 50,000 alcohol-dependent patients are actually receiving treatment intervention [3]. Excessive alcohol intake is a risk factor for various diseases, such as liver diseases [4], cardiovascular diseases [5], metabolic diseases [6], and malignancies [7,8], and it has been reported that alcohol intake is inversely related to prognosis [9]. According to published data from the Japan Society of Hepatology, although hepatitis C used to be the leading cause of cirrhosis, in recent years, alcoholic causes have overtaken hepatitis C as the leading cause [10]. A major paradigm shift is taking place in the area of liver cirrhosis in Japan.

The influence of alcohol on skeletal muscle is a recent topic [11]. Heavily drinking has adverse effects on skeletal muscle due to decreased food intake other than alcohol (i.e., starvation) [12], direct damage to skeletal muscle caused by acetaldehyde [13], decreased ammoniacal clearance [14], dysbiosis [15], gonadal hormone disorders [16,17], and hormonal growth disorders [18]. A negative correlation between endotoxin, a marker of leaky gut, and skeletal muscle mass (SMM) has been shown in male patients with alcoholic cirrhosis [15]. On the other hand, data from the United Kingdom (12,294 cases) show that SMM decreases with increasing alcohol consumption in both genders [19]. In that study, the majority of the subjects were in the 50–60 age range, and the authors opined that heavy daily drinking should be avoided in middle age and beyond [19].

However, to the best of our knowledge, large-scale clinical studies examining the relationship between alcohol intake and SMM in Japanese adults are rare. In order to clarify these matters, we decided to conduct a large-scale clinical study using data from Japanese health checkup recipients.

## 2. Patients and Methods

### 2.1. Our Study Subjects

Between September 2021 and November 2023, a total of 19,914 individual cases with available data on body composition and daily alcohol intake were identified in medical records and analyzed retrospectively. The amount of alcohol consumption was confirmed by means of a questionnaire. In all study cases, questionnaires were completed at our university hospital Health Sciences Clinic, with the procedure of body composition measurement conducted as previously reported [20]. In the present analysis, fat mass and fat-free (FF) mass (kg) were measured. The FF index was regarded as FF mass divided by height squared (kg/m^2^). In accordance with previous reports, SMM loss was regarded as an FF index <18 kg/m^2^ in males and an FF index <15 kg/m^2^ in females [21]. The fatty liver index (FLI) was calculated using waist circumference (WC), triglycerides (TGs), the body mass index (BMI), and gamma glutamyl transferase, as reported elsewhere [22].

### 2.2. Type Classification Based on Alcohol Consumed and Our Study Framework

In males, type A (never drinker) was defined as non-drinkers, type B (chance to mild drinker) as those who drank less than 210 g per week in terms of ethanol (30 g per day), type C (moderate drinker) as those who drank between 210 g and 420 g per week in terms of ethanol (30–60 g per day), and type D (severe drinker) as those who drank more than 420 g per week in terms of ethanol (60 g per day) [23]. In females, type A (never drinker) was defined as non-drinkers, type B (chance to mild drinker) as those who drank less than 140 g per week in terms of ethanol (20 g per day), type C (moderate drinker) as those who drank between 140 and 350 g per week in terms of ethanol (20–50 g per day), and type D (severe drinker) as those who drank more than 350 g per week in terms of ethanol (50 g per day) [23]. We retrospectively compared the FF index and other baseline features in the four types. This study conformed to the ethical guidelines of the Declaration of Helsinki, and approval was obtained from the ethics committee of OMPU hospital (approval no. 2023-159). This study was exempted from the requirement of written informed consent given the retrospective nature of the analysis. All data were analyzed anonymously.

### 2.3. Statistics

In the two-group comparison (continuous parameters), an unpaired *t*-test or Mann–Whitney *U*-test was adopted after estimating equal distribution. In the multiple-group comparison (continuous parameters), analysis of variance or the Kruskal–Wallis test was adopted after estimation of equal distribution. We applied Fisher’s exact test for the group comparison (nominal variables). Clinical data are shown as number or average (±standard deviation (SD)). A *p* value below 0.05 was considered to indicate a statistically significant difference by JMP 17.0.0 software (SAS Institute, Cary, NC, USA).

## 3. Results

### 3.1. Baseline Features

Baseline features in the current analyses are demonstrated in Table 1. The average (±SD) age was 52.2 ± 13.2 years in males (n = 8405) and 50.1 ± 12.1 years in females (n = 11,509) (*p* < 0.0001). The average (±SD) BMI was 23.8 ± 3.6 kg/m^2^ in males and 21.8 ± 3.7 kg/m^2^ in females (*p* < 0.0001). The average (±SD) WC was 85.2 ± 9.9 cm in males and 77.9 ± 9.8 cm in females (*p* < 0.0001). The average (±SD) FLI was 32.5 ± 26.0 in males and 14.2 ± 18.4 in females (*p* < 0.0001). The proportion of subjects of type A, B, C, and D was 36.5% (3064/8405), 44.2% (3716/8405), 5.9% (500/8405), and 13.4% (1125/8405), respectively, in males and 59.8% (6888/11,509), 31.3% (3607/11,509), 3.7% (424/11,509), and 5.1% (590/11,509), respectively, in females (*p* < 0.0001). The average (±SD) FF index was 18.5 ± 1.5 kg/m^2^ in males and 15.1 ± 1.0 kg/m^2^ in females (*p* < 0.0001). The proportion of subjects with decreased SMM in males (FF index <18 kg/m^2^) and females (FF index <15 kg/m^2^) was 39.0% (3280/8405) and 46.3% (5329/11,509), respectively. The proportion of subjects with 30 min or more exercise per day was 28.7% (2414/8405) in males and 18.5% (2132/11,509) in females (*p* < 0.0001). The average (±SD) fat mass was 15.7 ± 7.0 kg in males and 16.5 ± 7.2 kg in females (*p* < 0.0001).

### 3.2. Comparison of FF Index and the Proportion of Decreased SMM Among the Four Types in Males

**Figure 1:**
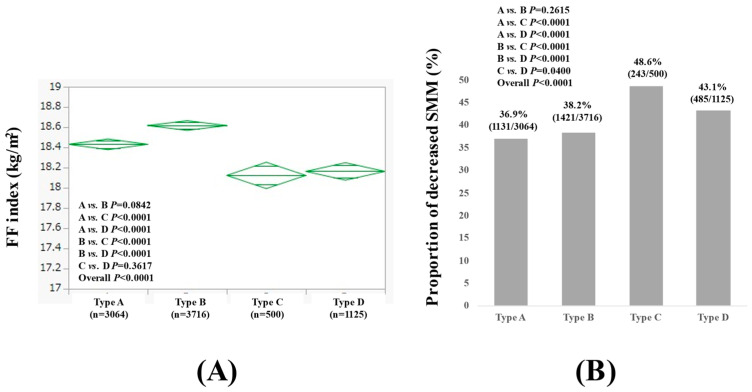
FF index (**A**) and the proportion of decreased skeletal muscle mass (SMM) (**B**) among four types in males (n = 8405). Type A was defined as non-drinkers, type B as those who drank less than 30 g per day in terms of ethanol, type C as those who drank between 30 and 60 g per day in terms of ethanol, and type D as those who drank more than 60 g per day in terms of ethanol.

The average (±SD) FF index in type A, B, C, and D males was 18.43 ± 1.37 kg/m^2^, 18.62 ± 1.61 kg/m^2^, 18.12 ± 1.02 kg/m^2^, and 18.16 ± 1.51 kg/m^2^, respectively (*p* values: comparison between A and B, *p* = 0.0842; A and C, *p* < 0.0001; A and D, *p* < 0.0001; B and C, *p* < 0.0001; B and D, *p* < 0.0001; C and D, *p* = 0.3617; overall, *p* < 0.0001) (Figure 1A). The proportion of decreased SMM in type A, B, C, and D males was 36.9% (1131/3064), 38.2% (1421/3716), 48.6% (243/500), and 43.1% (485/1125), respectively (*p* values: comparison between A and B, *p* = 0.2615; A and C, *p* < 0.0001; A and D, *p* < 0.0001; B and C, *p* < 0.0001; B and D, *p* < 0.0001; C and D, *p* = 0.040; overall, *p* < 0.0001) (Figure 1B). Comparison results for data other than the FF index among the four types of males are summarized in Table 2.

### 3.3. Comparison of FF Index and the Proportion of Decreased SMM Among Four Types in Females

**Figure 2:**
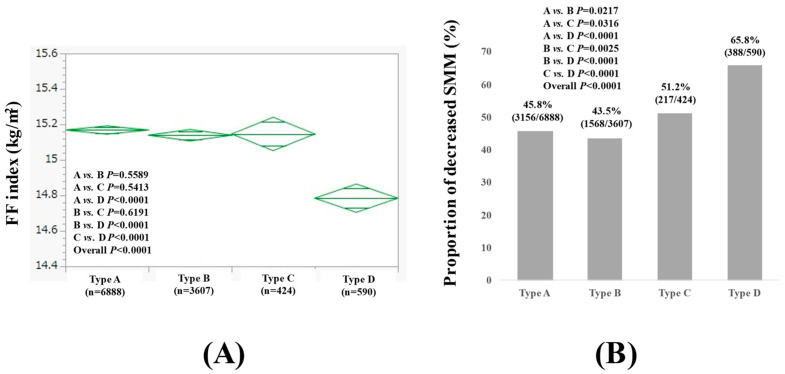
FF index (**A**) and the proportion of decreased skeletal muscle mass (SMM) (**B**) among four types of females (n = 11,509). Type A was defined as non-drinkers, type B as those who drank less than 20 g per day in terms of ethanol, type C as those who drank between 20 and 50 g per day in terms of ethanol, and type D as those who drank more than 50 g per day in terms of ethanol.

The average (±SD) FF index in type A, B, C, and D females was 15.17 ± 1.03 kg/m^2^, 15.14 ± 0.91 kg/m^2^, 15.15 ± 0.95 kg/m^2^, and 14.78 ± 0.95 kg/m^2^, respectively (*p* values: comparison between A and B, *p* = 0.5589; A and C, *p* = 0.5413; A and D, *p* < 0.0001; B and C, *p* = 0.6191; B and D, *p* < 0.0001; C and D, *p* < 0.0001; overall, *p* < 0.0001) (Figure 2A). The proportion of decreased SMM in type A, B, C, and D females was 45.8% (3156/6888), 43.5% (1568/3607), 51.2% (217/424), and 65.8% (388/590), respectively (*p* values: comparison between A and B, *p* = 0.0217; A and C, *p* = 0.0316; A and D, *p* < 0.0001; B and C, *p* = 0.0025; B and D, *p* < 0.0001; C and D, *p* < 0.0001; overall, *p* < 0.0001) (Figure 2B). Comparison results for data other than the FF index among the four types of females are summarized in Table 3.

### 3.4. Subgroup Analysis 1: Comparison of FF Index Among Four Types of Males (≥60 Years and <60 Years)

**Figure 3:**
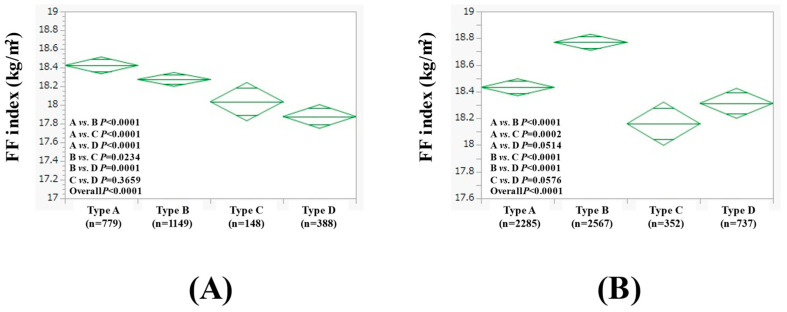
FF index among four types of males aged ≥60 years (n = 2464, (**A**)) and <60 years (n = 5941, (**B**)).

We also performed subgroup analyses stratified by age. In males aged ≥60 years (n = 2464), the average (±SD) FF index in the type A (n = 779), B (n = 1149), C (n = 148), and D (n = 388) groups was 18.42 ± 1.21 kg/m^2^, 18.27 ± 1.24 kg/m^2^, 18.04 ± 0.94 kg/m^2^, and 17.88 ± 1.56 kg/m^2^, respectively (*p* values: comparison between A and B, *p* < 0.0001; A and C, *p* < 0.0001; A and D, *p* < 0.0001; B and C, *p* = 0.0234; B and D, *p* = 0.0001; C and D, *p* = 0.3659; overall, *p* < 0.0001) (Figure 3A).

In males aged <60 years (n = 5941), the average (±SD) FF index in the type A (n = 2285), B (n = 2567), C (n = 352), and D (n = 737) groups was 18.43 ± 1.43 kg/m^2^, 18.77 ± 1.73 kg/m^2^, 18.16 ± 1.05 kg/m^2^, and 18.31 ± 1.46 kg/m^2^, respectively (*p* values: comparison between A and B, *p* < 0.0001; A and C, *p* = 0.0002; A and D, *p* = 0.0514; B and C, *p* < 0.0001; B and D, *p* < 0.0001; C and D, *p* = 0.0576; overall, *p* < 0.0001) (Figure 3B).

### 3.5. Subgroup Analysis 2: Comparison of FF Index Among Four Types of Females (≥60 Years and <60 Years)

**Figure 4:**
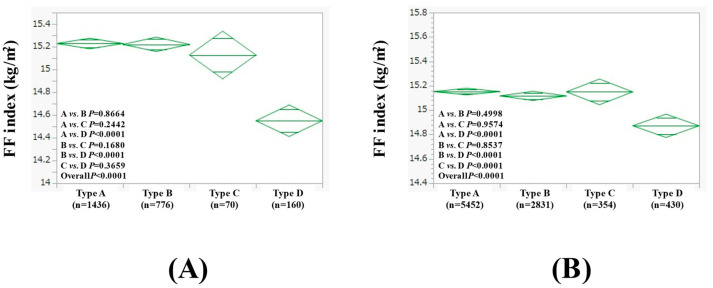
FF index among four types of females aged ≥60 years (n = 2442, (**A**)) and <60 years (n = 9067, (**B**)).

In females aged ≥60 years (n = 2442), the average (±SD) FF index in the type A (n = 1436), B (n = 776), C (n = 70), and D (n = 160) groups was 15.23 ± 0.95 kg/m^2^, 15.22 ± 0.83 kg/m^2^, 15.13 ± 0.81 kg/m^2^, and 14.55 ± 0.66 kg/m^2^, respectively (*p* values: comparison between A and B, *p* = 0.8664; A and C, *p* = 0.2442; A and D, *p* < 0.0001; B and C, *p* = 0.1680; B and D, *p* < 0.0001; C and D, *p* < 0.0001; overall, *p* < 0.0001) (Figure 4A).

In females aged <60 years (n = 9067), the average (±SD) FF index in the type A (n = 5452), B (n = 2831), C (n = 354), and D (n = 430) groups was 15.15 ± 1.05 kg/m^2^, 15.12 ± 0.94 kg/m^2^, 15.15 ± 0.97 kg/m^2^, and 14.87 ± 1.02 kg/m^2^, respectively (*p* values: comparison between A and B, *p* = 0.4998; A and C, *p* = 0.9574; A and D, *p* < 0.0001; B and C, *p* = 0.8537; B and D, *p* < 0.0001; C and D, *p* < 0.0001; overall, *p* < 0.0001) (Figure 4B).

### 3.6. Subgroup Analysis 3: Comparison of FF Index Among Four Types of Males (BMI ≥ 23 kg/m^2^ and BMI < 23 kg/m^2^)

**Figure 5:**
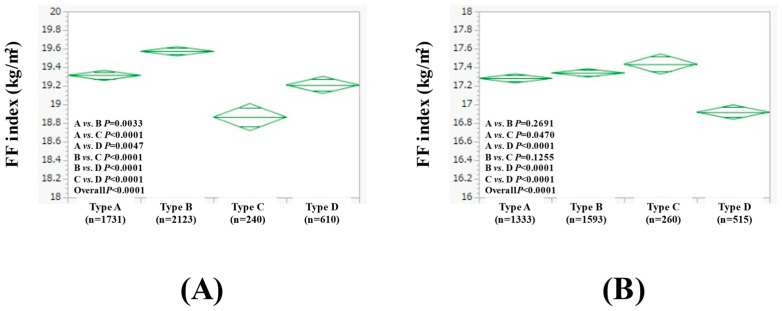
FF index among four types of males with BMI ≥ 23 kg/m^2^ (n = 4704, (**A**)) and BMI < 23 kg/m^2^ (n = 3701, (**B**)).

Furthermore, we performed subgroup analyses stratified by BMI. In males with BMI ≥ 23 kg/m^2^ (n = 4704), the average (±SD) FF index in the type A (n = 1731), B (n = 2123), C (n = 240), and D (n = 610) groups was 19.32 ± 0.93 kg/m^2^, 19.57 ± 1.37 kg/m^2^, 18.87 ± 0.73 kg/m^2^, and 19.21 ± 0.98 kg/m^2^, respectively (*p* values: comparison between A and B, *p* = 0.0033; A and C, *p* < 0.0001; A and D, *p* = 0.0047; B and C, *p* < 0.0001; B and D, *p* < 0.0001; C and D, *p* < 0.0001; overall, *p* < 0.0001) (Figure 5A).

In males with BMI < 23 kg/m^2^ (n = 3701), the average (±SD) FF index in the type A (n = 1333), B (n = 1593), C (n = 260), and D (n = 515) groups was 17.28 ± 0.94 kg/m^2^, 17.34 ± 0.84 kg/m^2^, 17.44 ± 0.72 kg/m^2^, and 16.92 ± 1.0 kg/m^2^, respectively (*p* values: comparison between A and B, *p* = 0.2691; A and C, *p* = 0.0470; A and D, *p* < 0.0001; B and C, *p* = 0.1255; B and D, *p* < 0.0001; C and D, *p* < 0.0001; overall, *p* < 0.0001) (Figure 5B).

### 3.7. Subgroup Analysis 4: Comparison of FF Index Among Four Types of Females (BMI ≥ 23 kg/m^2^ and BMI < 23 kg/m^2^)

**Figure 6:**
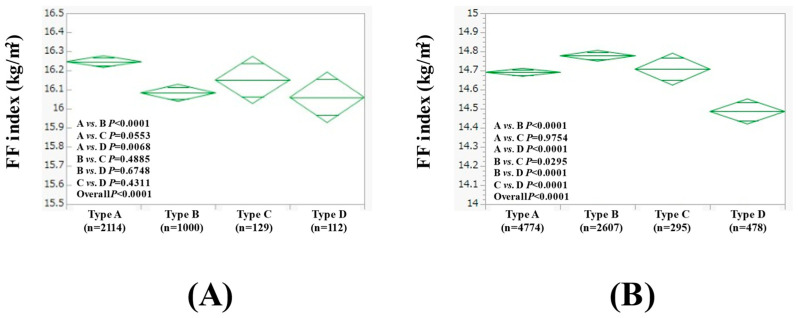
FF index among four types of females with BMI ≥ 23 kg/m^2^ (n = 3355, (**A**)) and BMI < 23 kg/m^2^ (n = 5941, (**B**)).

In females with BMI ≥ 23 kg/m^2^ (n = 3355), the average (±SD) FF index in the type A (n = 2114), B (n = 1000), C (n = 129), and D (n = 112) groups was 16.25 ± 0.75 kg/m^2^, 16.08 ± 0.64 kg/m^2^, 16.15 ± 0.72 kg/m^2^, and 16.06 ± 0.75 kg/m^2^, respectively (*p* values: comparison between A and B, *p* < 0.0001; A and C, *p* = 0.0553; A and D, *p* = 0.0068; B and C, *p* = 0.4885; B and D, *p* = 0.6748; C and D, *p* = 0.4311; overall, *p* < 0.0001) (Figure 6A).

In females with BMI < 23 kg/m^2^ (n = 8154), the average (±SD) FF index in the type A (n = 4774), B (n = 2607), C (n = 295), and D (n = 478) groups was 14.69 ± 0.74 kg/m^2^, 14.78 ± 0.73 kg/m^2^, 14.71 ± 0.65 kg/m^2^, and 14.49 ± 0.71 kg/m^2^, respectively (*p* values: comparison between A and B, *p* < 0.0001; A and C, *p* = 0.9754; A and D, *p* < 0.0001; B and C, *p* = 0.0295; B and D, *p* < 0.0001; C and D, *p* < 0.0001; overall, *p* < 0.0001) (Figure 6B).

## 4. Discussion

While the tradition and culture of alcohol are deeply rooted in the lives of the people, inappropriate drinking can lead to serious health problems. The Japanese Ministry of Health, Labour and Welfare (MHLW) recently issued “Guidelines on Health-Conscious Drinking”, noting that heavy drinking increases the risk of sarcopenia [2]. However, detailed data on alcohol consumption and SMM in the Japanese population are currently scarce. For Japanese who prefer to drink, abstinence from alcohol can be psychologically stressful. Is not drinking alcohol at all recommended in terms of skeletal muscle maintenance, or is a certain amount of alcohol consumption acceptable? This point seems to require discussion. To clarify these matters, the present large-scale clinical study was conducted.

In the analysis of the overall cases, the FF index was relatively higher for type B than for type A males, and the FF index was markedly lower for type C and D males. In females, there were no significant differences between types A, B, and C, and the FF index was significantly lower in type D females. These results indicate that at least habitual heavy drinking has a negative effect on skeletal muscle. However, from the standpoint of maintaining SMM, it is not necessary to completely abstain from alcohol. Our study results are consistent with a previous report [19]. While the concept of “harm reduction” by reducing the amount of alcohol consumption has been recently proposed [24]. A brief intervention (BI) is a psychological intervention consisting of six items (feedback, responsibility, advice, menu, empathy, and self-efficacy) aimed at reducing alcohol consumption [25]. Significant improvements in Alcohol Use Disorders Identification Test scores have been reported in habitual drinkers receiving BI [26]. Wilk et al. also reviewed 12 randomized controlled trials and concluded that “drinkers who received BI were twice as likely to reduce their alcohol intake over 6 to 12 months period than those without receiving BI, and BI for heavy drinkers is an effective and low-cost preventive measure” [27]. On the other hand, nalmefene is an alcohol-reducing drug that suppresses the desire to drink by acting on opioid receptors widely distributed in the central nervous system [28]. In a phase III clinical trial of nalmefene, study subjects were categorized into three groups: nalmefene 10 mg/day, 20 mg/day, and control, each administered for 24 weeks. In the primary outcome measure of change (baseline to 12 weeks) of the number of days of heavy drinking, there was a noted difference between the 10 mg nalmefene group and the 20 mg nalmefene group compared with the control (*p* < 0.0001), and the effect was maintained until the end of the 24-week treatment period. An important secondary outcome measure, total alcohol consumption, was also significantly reduced in both nalmefene-treated groups (*p* < 0.0001) [29]. The efficacy and safety of long-term nalmefene therapy (48 weeks) have also been reported [30]. In view of these reports, BI and sobriety medications may be required, especially in type D patients. As noted above, complete abstinence from alcohol is not necessary to maintain SMM. Small amounts of alcohol consumption increase SMM and improve prognosis [9,19]. The Japanese MHLW states that the amounts that increases the risk of lifestyle-related diseases is a daily net alcohol intake of 40 g or more and 20 g or more for men and women, respectively [2]. Mild to moderate alcohol consumption does not have a negative effect on SMM.

In a comparison of FF index between type A and B in all cases, the FF index in type B tended to increase more than that in type A men, while there was no significant difference in women. Given the results of the stratified analysis, one factor contributing to these results is that in men, the subgroups under age 60 and with a BMI over 23 kg/m^2^ are more physically active in type B. The percentage of patients who exercised at least 30 min per day was 24.3% (554/2285) for type A and 27.2% (697/2567) for type B in the group of men younger than 60 years and 24.9% (431/1731) for type A and 29.8% (633/2123) for type B in the group with BMI ≥ 23 kg/m^2^, with a higher rate for type B in both subgroups; however, no such trend was found in women. In this study, fat mass was significantly higher in females (male fat mass (average ± SD): 15.7 ± 7.0 kg; female fat mass (average ± SD): 16.5 ± 7.2 kg, *p* < 0.0001), despite a significantly higher BMI in males (male BMI (average ± SD): 23.8 ± 3.6 kg/m^2^; female BMI (average ± SD): 21.8 ± 3.7 kg/m^2^, *p* < 0.0001). In general, females are more inclined to store a significant amount of fat in reserves, which is used to generate energy rather than fat from skeletal muscle reserves, which may make females more resistant to muscle wasting as compared with males [31]. These facts are relevant to the differences in results between men and women in this study. Type D females had the lowest results among the four groups, not only in FF index but also in fat mass, possibly due to lower dietary intake associated with heavy alcohol consumption [12]. As shown in Figure 3A, in male subjects aged 60 years or older, a trend toward lower SMM in response to alcohol intake was observed, which is in line with previous reports [19]. On the other hand, alcohol causes various metabolic disorders [32,33,34], which may be indicated by the results of TG, FLI, fasting blood glucose, blood pressure, etc., in this study.

It is necessary to mention the limitations of this study. First, this study involved a single-center, retrospective, and cross-sectional analysis. Second, the duration of alcohol intake in each study subject was unclear, leading to bias. Third, this study was limited to the Japanese population, so it is unclear whether the results hold true for other racial groups. Fourth, the daily alcohol intake was calculated based on self-reports, and baseline factors in the four groups (type A, B, C, and D) were largely different, as shown in Table 2 and Table 3, also creating bias. Therefore, the results should be interpreted with caution. However, this study found that in Japanese adults, at least habitual heavily drinking has a negative effect on skeletal muscle—but not to the extent that complete abstinence from alcohol is necessary in terms of maintaining SMM. Finally, the authors would like to emphasize that alcohol consumption is not necessarily harmful in terms of maintaining SMM, as long as the amount consumed is kept at a mild level.

## 5. Conclusions

Habitual heavily drinking has a negative effect on skeletal muscle, but it is not necessary to completely abstain from drinking in terms of maintaining SMM.

## Figures and Tables

**Table 1:** Baseline features (n = 19,914).

	Male (n = 8405)	Female (n = 11,509)	Overall *p* Value
Age (years)	52.2 ± 13.2	50.1 ± 12.1	<0.0001
BMI (kg/m^2^)	23.8 ± 3.6	21.8 ± 3.7	<0.0001
Type A/B/C/D	3064(36.5%)/3716(44.2%)/ 500(5.9%)/1125(13.4%)	6888(59.8%)/3607(31.3%)/ 424(3.7%)/590(5.1%)	<0.0001
WC (cm)	85.2 ± 9.9	77.9 ± 9.8	<0.0001
30 min or more exercise per day, yes/no/unknown	2414(28.7%)/5636(67.1%) /355(4.2%)	2132(18.5%)/9037(78.5%) /340(3.0%)	<0.0001
Hemoglobin (g/dL)	14.9 ± 1.1	13.1 ± 1.1	<0.0001
ALT (IU/L)	26.9 ± 19.5	17.3 ± 11.7	<0.0001
GGT (IU/L)	45.2 ± 53.0	23.6 ± 27.6	<0.0001
Serum albumin (g/dL)	4.4 ± 0.3	4.3 ± 0.2	<0.0001
Triglycerides (mg/dL)	118.7 ± 89.6	80.5 ± 50.3	<0.0001
Fatty liver index	32.5 ± 26.0	14.2 ± 18.4	<0.0001
FBS (mg/dL)	95.9 ± 19.8	88.5 ± 12.5	<0.0001
Uric acid (mg/dL)	6.2 ± 1.3	4.6 ± 1.0	<0.0001
eGFR (ml/min/1.73 m^2^)	70.9 ± 13.5	74.1 ± 13.6	<0.0001
sBP (mmHg)	122.5 ± 16.7	115.3 ± 17.4	<0.0001
dBP (mmHg)	78.7 ± 12.6	71.6 ± 12.1	<0.0001
Fat mass (kg)	15.7 ± 7.0	16.5 ± 7.2	<0.0001
FF index (kg/m^2^)	18.5 ± 1.5	15.1 ± 1.0	<0.0001

Data are shown as number or average (±standard deviation). BMI, body mass index; WC, waist circumference; ALT, alanine aminotransferase; GGT, gamma glutamyl transferase; FBS, fasting blood sugar; eGFR, estimated glomerular filtration rate; sBP, systolic blood pressure; dBP, diastolic blood pressure; FF index, fat-free mass divided by height squared.

**Table 2:** Results for comparison of baseline data other than FF index among the four types of males.

Males (n = 8405)	Type A (n = 3064)	Type B (n = 3716)	Type C (n = 500)	Type D (n = 1125)	Overall *p* Value
Age (years)	50.5 ± 13.9	52.7 ± 12.7	52.8 ± 13.7	54.9 ± 12.0	<0.0001
BMI (kg/m^2^)	23.7 ± 3.3	24.2 ± 4.1	23.0 ± 2.3	23.4 ± 3.3	<0.0001
30 min or more exercise per day, yes/no/unknown	779(25.4%)/2141 (69.9%)/144(4.7%)	1144(30.8%)/2473 (66.6%)/99(2.7%)	168(33.6%)/288 (57.6%)/44(8.8%)	323(28.7%)/734 (65.2%)/68(6.0%)	<0.0001
WC (cm)	84.5 ± 9.5	86.1 ± 10.6	83.2 ± 7.5	85.0 ± 9.0	<0.0001
Hemoglobin (g/dL)	14.9 ± 1.1	14.9 ± 1.1	14.7 ± 1.1	14.8 ± 1.2	0.0011
ALT (IU/L)	26.9 ± 18.8	27.4 ± 20.9	24.1 ± 15.0	26.7 ± 18.3	0.0249
GGT (IU/L)	32.3 ± 27.4	44.8 ± 47.5	52.3 ± 70.5	78.6 ± 88.1	<0.0001
Serum albumin (g/dL)	4.4 ± 0.3	4.4 ± 0.3	4.4 ± 0.3	4.3 ± 0.3	0.0018
Triglycerides (mg/dL)	111.6 ± 81.8	119.2 ± 86.8	120.0 ± 91.2	136.1 ± 113.2	<0.0001
Fatty liver index	28.5 ± 23.7	34.3 ± 27.1	29.2 ± 23.8	38.9 ± 27.6	<0.0001
FBS (mg/dL)	94.6 ± 20.1	96.3 ± 19.0	96.4 ± 19.8	97.6 ± 19.4	<0.0001
Uric acid (mg/dL)	6.0 ± 1.2	6.3 ± 1.2	6.3 ± 1.3	6.4 ± 1.3	<0.0001
eGFR (ml/min/1.73 m^2^)	70.7 ± 14.0	70.6 ± 13.2	71.2 ± 13.4	72.3 ± 13.4	0.0032
Fat mass (kg)	15.3 ± 6.2	16.3 ± 8.0	14.2 ± 4.7	15.3 ± 6.0	<0.0001
sBP (mmHg)	119.7 ± 16.4	123.3 ± 16.3	123.1 ± 16.3	127.6 ± 17.5	<0.0001
dBP (mmHg)	76.1 ± 12.6	79.4 ± 12.3	79.6 ± 12.0	82.6 ± 12.6	<0.0001

Data are shown as number or average (±standard deviation). BMI, body mass index; WC, waist circumference; ALT, alanine aminotransferase; GGT, gamma glutamyl transferase; FBS, fasting blood sugar; eGFR, estimated glomerular filtration rate; sBP, systolic blood pressure; dBP, diastolic blood pressure; FF index, fat-free mass divided by height squared.

**Table 3:** Results for comparison of baseline data other than FF index among the four types of females.

Females (n = 11,509)	Type A (n = 6888)	Type B (n = 3607)	Type C (n = 424)	Type D (n = 590)	Overall *p* Value
Age (years)	49.8 ± 12.1	50.4 ± 11.9	49.3 ± 11.2	52.6 ± 13.7	<0.0001
BMI (kg/m^2^)	21.9 ± 3.8	21.6 ± 3.3	21.9 ± 3.7	20.8 ± 3.2	<0.0001
30 min or more exercise per day, yes/no/unknown	1249(18.1%)/5505 (79.9%)/134(1.9%)	688(19.1%)/2800 (77.6%)/119(33.0%)	92(21.7%)/319 (75.2%)/13(3.1%)	103(17.5%)/413 (70.0%)/74(12.5%)	<0.0001
WC (cm)	78.2 ± 10.1	77.6 ± 9.3	78.2 ± 9.8	77.0 ± 8.8	0.0287
Hemoglobin (g/dL)	13.0 ± 1.2	13.1 ± 1.1	13.1 ± 1.1	13.0 ± 1.3	0.7007
ALT (IU/L)	17.6 ± 12.2	16.8 ± 10.3	17.4 ± 13.6	17.6 ± 11.8	0.5021
GGT (IU/L)	21.6 ± 20.5	23.9 ± 21.1	31.1 ± 41.4	39.4 ± 75.5	<0.0001
Serum albumin (g/dL)	4.3 ± 0.2	4.3 ± 0.2	4.3 ± 0.2	4.3 ± 0.2	0.075
Triglycerides (mg/dL)	80.3 ± 46.8	78.5 ± 45.4	81.9 ± 44.8	94.1 ± 97.2	0.0002
Fatty liver index	14.5 ± 19.1	13.3 ± 16.8	16.0 ± 19.9	15.2 ± 19.0	0.0002
FBS (mg/dL)	88.2 ± 13.2	88.6 ± 11.4	89.3 ± 10.0	90.0 ± 12.8	<0.0001
Uric acid (mg/dL)	4.6 ± 1.0	4.7 ± 1.0	4.8 ± 1.0	5.0 ± 1.2	<0.0001
eGFR (mL/min/1.73 m^2^)	73.9 ± 13.4	73.7 ± 13.6	75.7 ± 13.8	76.4 ± 15.0	<0.0001
Fat mass (kg)	16.8 ± 7.5	16.1 ± 6.5	16.9 ± 7.6	15.1 ± 6.5	<0.0001
sBP (mmHg)	114.8 ± 17.4	115.3 ± 17.2	117.6 ± 17.1	119.5 ± 18.0	<0.0001
dBP (mmHg)	71.1 ± 11.9	71.8 ± 11.9	74.2 ± 12.7	74.6 ± 13.1	<0.0001

Data are shown as number or as average (±standard deviation). BMI, body mass index; WC, waist circumference; ALT, alanine aminotransferase; GGT, gamma glutamyl transferase; FBS, fasting blood sugar; eGFR, estimated glomerular filtration rate; sBP, systolic blood pressure; dBP, diastolic blood pressure; FF index, fat-free mass divided by height squared.

## Data Availability

Data are available upon request due to restrictions, e.g., privacy or ethical considerations.

## Abbreviations

SMM: skeletal muscle mass; FF mass: fat-free mass; FLI: fatty liver index; BMI: body mass index; TG: triglyceride; WC: waist circumference; SD: standard deviation; MHLW: Ministry of Health, Labour and Welfare; BI: brief intervention.

## References

[1] Yamamuro B. (1968). Origins of some Japanese drinking customs. Q. J. Stud. Alcohol.

[2] https://www.mhlw.go.jp/stf/newpage_38541.html.

[3] Osaki Y., Kinjo A., Higuchi S., Matsumoto H., Yuzuriha T., Horie Y., Kimura M., Kanda H., Yoshimoto H. (2016). Prevalence and Trends in Alcohol Dependence and Alcohol Use Disorders in Japanese Adults; Results from Periodical Nationwide Surveys. Alcohol Alcohol..

[4] Hanai T., Nishimura K., Unome S., Miwa T., Nakahata Y., Imai K., Suetsugu A., Takai K., Shimizu M. (2024). Alcohol-associated liver disease increases the risk of muscle loss and mortality in patients with cirrhosis. J. Gastroenterol..

[5] Krittanawong C., Isath A., Rosenson R.S., Khawaja M., Wang Z., Fogg S.E., Virani S.S., Qi L., Cao Y., Long M.T. (2022). Alcohol Consumption and Cardiovascular Health. Am. J. Med..

[6] Llamosas-Falcón L., Rehm J., Bright S., Buckley C., Carr T., Kilian C., Lasserre A.M., Lemp J.M., Zhu Y., Probst C. (2023). The Relationship Between Alcohol Consumption, BMI, and Type 2 Diabetes: A Systematic Review and Dose-Response Meta-analysis. Diabetes Care.

[7] Bagnardi V., Rota M., Botteri E., Tramacere I., Islami F., Fedirko V., Scotti L., Jenab M., Turati F., Pasquali E. (2015). Alcohol consumption and site-specific cancer risk: A comprehensive dose-response meta-analysis. Br. J. Cancer.

[8] Rumgay H., Murphy N., Ferrari P., Soerjomataram I. (2021). Alcohol and Cancer: Epidemiology and Biological Mechanisms. Nutrients.

[9] Day E., Rudd J.H.F. (2019). Alcohol use disorders and the heart. Addiction.

[10] Enomoto H., Akuta N., Hikita H., Suda G., Inoue J., Tamaki N., Ito K., Akahane T., Kawaoka T., Morishita A. (2024). Etiological changes of liver cirrhosis and hepatocellular carcinoma-complicated liver cirrhosis in Japan: Updated nationwide survey from 2018 to 2021. Hepatol. Res..

[11] Simon L., Bourgeois B.L., Molina P.E. (2023). Alcohol and Skeletal Muscle in Health and Disease. Alcohol. Res..

[12] Dasarathy J., McCullough A.J., Dasarathy S. (2017). Sarcopenia in Alcoholic Liver Disease: Clinical and Molecular Advances. Alcohol. Clin. Exp. Res..

[13] Oba T., Maeno Y., Ishida K. (2005). Differential contribution of clinical amounts of acetaldehyde to skeletal and cardiac muscle dysfunction in alcoholic myopathy. Curr. Pharm. Des..

[14] Kant S., Davuluri G., Alchirazi K.A., Welch N., Heit C., Kumar A., Gangadhariah M., Kim A., McMullen M.R., Willard B. (2019). Ethanol sensitizes skeletal muscle to ammonia-induced molecular perturbations. J. Biol. Chem..

[15] Sato S., Namisaki T., Murata K., Fujimoto Y., Takeda S., Enomoto M., Shibamoto A., Ishida K., Ogawa H., Takagi H. (2021). The association between sarcopenia and endotoxin in patients with alcoholic cirrhosis. Medicine.

[16] Grossmann M., Hoermann R., Gani L., Chan I., Cheung A., Gow P.J., Li A., Zajac J.D., Angus P. (2012). Low testosterone levels as an independent predictor of mortality in men with chronic liver disease. Clin. Endocrinol..

[17] Sinclair M., Grossmann M., Hoermann R., Angus P.W., Gow P.J. (2016). Testosterone therapy increases muscle mass in men with cirrhosis and low testosterone: A randomised controlled trial. J. Hepatol..

[18] Tentler J.J., LaPaglia N., Steiner J., Williams D., Castelli M., Kelley M.R., Emanuele N.V., Emanuele M.A. (1997). Ethanol, growth hormone and testosterone in peripubertal rats. J. Endocrinol..

[19] Skinner J., Shepstone L., Hickson M., Welch A.A. (2023). Alcohol Consumption and Measures of Sarcopenic Muscle Risk: Cross-Sectional and Prospective Associations Within the UK Biobank Study. Calcif. Tissue Int..

[20] Onishi S., Fukuda A., Matsui M., Ushiro K., Nishikawa T., Asai A., Kim S.K., Nishikawa H. (2023). Body Composition Analysis in Patients with Metabolic Dysfunction-Associated Fatty Liver Disease. Nutrients.

[21] Kawakami R., Tanisawa K., Ito T., Usui C., Miyachi M., Torii S., Midorikawa T., Ishii K., Muraoka I., Suzuki K. (2022). Fat-Free Mass Index as a Surrogate Marker of Appendicular Skeletal Muscle Mass Index for Low Muscle Mass Screening in Sarcopenia. J. Am. Med. Dir. Assoc..

[22] Ushiro K., Matsui M., Fukuda A., Onishi S., Nishikawa T., Asai A., Kim S.K., Nishikawa H. (2024). Fatty liver index and somatic composition in subjects receiving medical health checkup. Hepatol. Res..

[23] European Association for the Study of the Liver (EASL), European Association for the Study of Diabetes (EASD), European Association for the Study of Obesity (EASO) (2024). EASL-EASD-EASO Clinical Practice Guidelines on the management of metabolic dysfunction-associated steatotic liver disease (MASLD). J. Hepatol..

[24] Crowther D., Curran J., Somerville M., Sinclair D., Wozney L., MacPhee S., Rose A.E., Boulos L., Caudrella A. (2023). Harm reduction strategies in acute care for people who use alcohol and/or drugs: A scoping review. PLoS ONE.

[25] Hammock K., Velasquez M.M., Alwan H., von Sternberg K. (2020). Alcohol Screening, Brief Intervention, and Referral to Treatment (SBIRT) for Girls and Women. Alcohol. Res..

[26] Hara N., Hiraoka A., Nakai M., Shiraki M., Namisaki T., Miyaaki H., Hisanaga T., Takahashi H., Ohama H., Tada F. (2024). Brief intervention for chronic liver disease patients with alcohol use disorder in a hepatology outpatient unit: Effects and limitations. Hepatol. Res..

[27] Wilk A., Jensen N., Havighurst T. (1997). Meta-analysis of randomized control trials addressing brief interventions in heavy alcohol drinkers. J. Gen. Intern. Med..

[28] Palpacuer C., Duprez R., Huneau A., Locher C., Boussageon R., Laviolle B., Naudet F. (2018). Pharmacologically controlled drinking in the treatment of alcohol dependence or alcohol use disorders: A systematic review with direct and network meta-analyses on nalmefene, naltrexone, acamprosate, baclofen and topiramate. Addiction.

[29] Miyata H., Takahashi M., Murai Y., Tsuneyoshi K., Hayashi T., Meulien D., Sørensen P., Higuchi S. (2019). Nalmefene in alcohol-dependent patients with a high drinking risk: Randomized controlled trial. Psychiatry Clin. Neurosci..

[30] Higuchi S., Takahashi M., Murai Y., Tsuneyoshi K., Nakamura I., Meulien D., Miyata H. (2020). Long-term safety and efficacy of nalmefene in Japanese patients with alcohol dependence. Psychiatry Clin. Neurosci..

[31] Ko S.H., Jung Y. (2021). Energy Metabolism Changes and Dysregulated Lipid Metabolism in Postmenopausal Women. Nutrients.

[32] You M., Arteel G.E. (2019). Effect of ethanol on lipid metabolism. J. Hepatol..

[33] Aberg F., Byrne C.D., Pirola C.J., Männistö V., Sookoian S. (2023). Alcohol consumption and metabolic syndrome: Clinical and epidemiological impact on liver disease. J. Hepatol..

[34] Fuchs F.D., Fuchs S.C. (2021). The Effect of Alcohol on Blood Pressure and Hypertension. Curr. Hypertens. Rep..

